# A case report and literature review on giant tumefactive perivascular spaces

**DOI:** 10.3389/fnins.2025.1554910

**Published:** 2025-04-09

**Authors:** Chang Cheng, Yiqi Pan, Ke Ma, Shuhan Liu, Xiaoli Mai

**Affiliations:** ^1^Department of Radiology, Nanjing Drum Tower Hospital, Affiliated Hospital of Medical School, Nanjing University, Nanjing, China; ^2^Department of Radiology, Nanjing Drum Tower Hospital Clinical College of Nanjing Medical University, Nanjing, China; ^3^Department of Radiology, Nanjing Drum Tower Hospital Clinical College of Xuzhou Medical University, Nanjing, China

**Keywords:** giant tumefactive perivascular spaces, perivascular spaces, lymphatic system, magnetic resonance imaging, case diagnosis

## Abstract

**Background:**

Perivascular spaces (PVS) are fluid-filled cavities located in the brain that surround blood vessels. Dilated PVS (dPVS) can be discerned on MRI in healthy individuals. Extreme expansion of PVS is classified as giant tumefactive PVS (GTPVS), a rare condition with an ambiguous etiology. Although GTPVS may exert a mass effect, it should not be misidentified as a tumor or other pathological conditions.

**Case presentation:**

We report a rare case of GTPVS that was incidentally discovered during the examination of a scalp mass. The patient exhibited giant tumefactive dilation of PVS in the left cerebral hemisphere without any clinical symptoms. The MR imaging of this case showed a typical cluster cyst. Based on the location of the lesion, consider it belongs to Type II GTPVS.

**Conclusion:**

This study reports a rare GTPVS case, establishing diagnostic criteria, differential diagnosis, and management strategies. While asymptomatic cases require no treatment, hydrocephalus may necessitate surgery. Literature review suggests PVS dilation reflects glymphatic dysfunction, providing new pathophysiological insights. Further studies are needed to validate these findings due to the condition’s rarity.

## Introduction

Perivascular spaces (PVS) are fluid-filled compartments in the brain that travel along blood vessels (Kwee and Kwee, 2007). A PVS diameter of less than 2 mm is considered normal, while dilated PVS (dPVS) can be observed on MRI in healthy individuals. Extreme dilation of PVS is termed giant tumefactive PVS (GTPVS). The dilated PVS is located in the basal ganglia along the beanstalk artery (type I), subcortical white matter along the perforating medullary artery (type II) and midbrain (type III) (Benson et al., 2024). GTPVS usually presents as a cluster of cystic structures larger than 1.5 cm (Al Abdulsalam et al., 2018), exhibiting an iso-signal relative to cerebrospinal fluid (CSF) and a lack of enhancement on imaging studies. These structures are most commonly located in the midbrain thalamic region.

Patients with GTPVS usually have no significant clinical manifestations, but there may be instances of cognitive impairment or hydrocephalus (Woo et al., 2018). Ventricular drainage may be necessary if hydrocephalus is present.

## Case presentation

A 72-year-old male presented to our hospital complaining of discomfort and a lump on his scalp, with no notable medical or surgical history.

**FIGURE 1:**
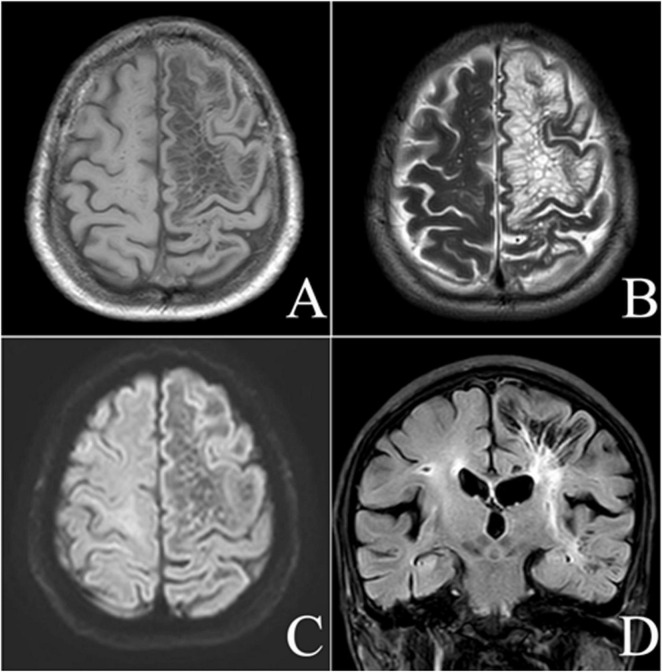
Male, 72-year-old, discomfort and a scalp mass for more than 4 months. MRI axial T1-weighted **(A)**, axial T2-weighted **(B)**, diffusion-weighted **(C)**, and coronal fluid-attenuated inversion recovery-weighted **(D)** images showing a giant tumefactive perivascular space (GTPVS) located in the left cerebral hemisphere, which shows isointense relative to cerebrospinal fluid (CSF). White matter high signal can be seen surround the lesion. Cavity foci are identified in the right radiocoronal area and adjacent to the lesion.

The intracranial manifestation of the lesion was situated within the left cerebral hemisphere, predominantly affecting the left frontoparietal lobe, affects almost all the white matter areas located in the subcortical region. This lesion is characterized by its clustered distribution and well-demarcated borders. It exhibited a CSF signal, with hypointensity on T1-weighted images (T1WI), T2-fluid attenuated inversion recovery (FLAIR), and hyperintensity on T2-weighted images (T2WI). Additionally, there was an increased FLAIR signal around the lesion, which involved the left cerebral hemisphere. Apart from the primary lesion, the intracranial region also exhibits areas of white matter hyperintensity on FLAIR and lacunar infarcts. Based on these findings, this case is categorized as belonging to a specific type (Figure 1).

**FIGURE 2:**
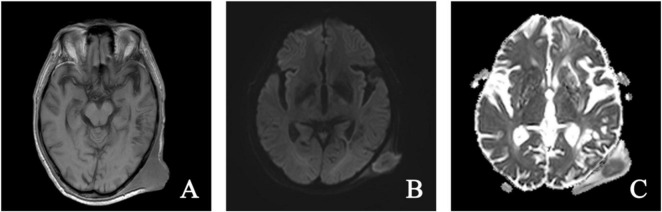
Magnetic resonance imaging axial T1-weighted **(A)**, diffusion-weighted **(B)**, and apparent diffusion coefficient **(C)** images. On T1-weighted imaging, a slightly hyperintense, round-shaped lesion was observed in the subcutaneous region of the left occipital area. The lesion exhibited high signal intensity on DWI and low signal intensity on the ADC. The internal signal of the lesion appeared heterogeneous, with an approximate size of 31 × 28 mm.

**FIGURE 3:**
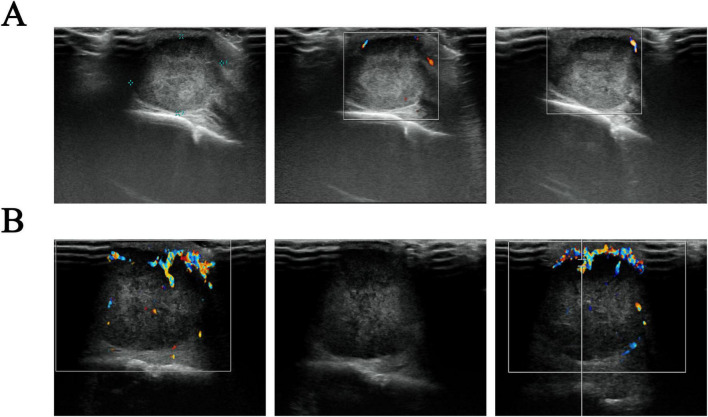
Ultrasound images of a scalp mass. **(A)** The initial assessment reveals a sub-scalp hypoechoic mass, about 21 × 15 mm in extent. This mass exhibits a defined envelope, regular morphology, and clear boundary, accompanied by uneven internal echogenicity. Color Doppler Flow Imaging (CDFI) indicated no obvious blood flow signal; **(B)** a re-examination conducted 2 months later suggests that the sub-scalp hypoechoic mass has increased in size to approximately 32 × 24 × 26 mm. The mass now lacks an envelope but still presents with regular morphology and clear boundaries, as well as uneven internal echogenicity. CDFI showed increased blood flow signals both in the periphery and within the mass, characterized by a harmonic frequency pattern indicative of an arterial spectrum.

The scalp mass was located in the galeal aponeurotic layer of the scalp, displaying isointensity on T1WI and T2WI, with slight hyperintensity on FLAIR and diffusion weighted imaging (DWI) (Figure 2). Color Doppler Flow Imaging (CDFI) indicated a shadow of blood flow around the lesion (Figure 3). Based on these findings, we hypothesize that the mass was a sebaceous cyst complicated by infection.

## Discussion

The boundary of the PVS is demarcated by the endothelial basement membranes and the glial limitans of the parenchymal perforating vessels, which are continuous with the capillaries’ basal lamina (Klostranec et al., 2021). As small arteries branch into capillaries, the occipital sheath basement membranes and astrocytes (i.e., glial cells) interconnect to form the PVS (Agarwal and Carare, 2020). GTPVSs are considered as extremely swollen PVS.

GTPVS can be classified into three distinct anatomical types: Type I follows the lenticulostriate artery through the anterior perforating substance into the basal ganglia region; Type II is associated with the trajectory of the medullary artery, extending into the cortical gray matter through the high convexity and into the white matter; and Type III is located in the mesencephalon thalamic region (Kwee and Kwee, 2019), with the most common being Type III. It may be related to the fact that type III is often associated with obstructive hydrocephalus and causes symptoms. GTPVS are rare (Table 1), with only about 20 of them exhibiting enlargement similar to that seen in this study (Al Abdulsalam et al., 2018; Woo et al., 2018). The patient in this case belonged to type II with typical imaging manifestations.

**TABLE 1:** Typology of GTPVS in the literature (Kwee and Kwee, 2019).

Classification	Number of cases	Presenting symptoms
Type I	5	2 (2 patients are not specified)
Type II	62	8 (29 patients are not specified or applicable)
Type III	80	52 (27 patients are not specified or applicable)

The clinical manifestations of GTPVS vary depending on their occupying effect, location and degree of cyst expansion, with approximately 50% of GTPVS patients having headaches (Al Abdulsalam et al., 2018). Additionally, some GTPVSs may present with localized neurological symptoms in the midbrain such as hemiparesis, cerebral tremor and motor nerve palsy (Woo et al., 2018). Besides, GTPVS–particularly Type II GTPVS–may also present with concomitant cognitive dysfunction. This phenomenon may be attributed to two potential mechanisms: firstly, Type II lesions are frequently associated with white matter hyperintensities (Buerge et al., 2011; Woo et al., 2018), as observed in our case; secondly, it may be related to dysfunction of the intracranial lymphatic system.

Although the etiology of PVS dilatation remains unclear, some hypotheses suggest that PVS dilation may occur secondary to ischemic injury to the brain parenchyma due to microvascular disease or ex vacuo periarteriolar changes, leading to interstitial fluid (ISF) leakage, increased vascular permeability, and fluid accumulation (Charidimou et al., 2013). Nevertheless, the current study indicates that the PVS fulfills several important functions: (1) it acts the main channel for the outflow of ISF from and to the brain parenchyma; (2) it facilitates the transport of various signaling molecules; (3) it plays a vital role in the exchange of ISF with CSF in the central nervous system (CNS); and (4) it aids in the elimination of neurological waste products, contributing to the regulation of fluid circulation within the brain (Nedergaard, 2013). Recent studies also suggest a lymphatic-like system transports CSF/solutes via periarterial pathways and clears ISF/solutes through perivenous routes, potentially connected to extracranial veins via emissary veins (Iliff et al., 2012; Louveau et al., 2017). Thus a dilation of PVS may suggest a deterioration in the function of the intracerebral lymphatic system (Nedergaard, 2013), which may be associated with cognitive function and the development of cerebrovascular disease (Iliff et al., 2012). In this case, the subcutaneous scalp mass was identified within the galeal aponeurotic layer. Based on the aforementioned hypotheses, we propose that the mass may have compromised venous and lymphatic drainage by compressing the draining veins and lymphatic vessels within the subgaleal space. This mechanism may explain the pronounced swelling observed.

MRI features of PVS help to differentiate enlarged PVS from other cystic brain lesions are crucial for distinguishing enlarged PVS from other cystic brain lesions such as low-grade gliomas and parasitic infections. GTPVSs sometimes mimic a variety of pathological processes, including cystic tumors, infectious or parasitic cysts, neuroepithelial cysts, cavernous cystic infarcts, cystic white matter softening and mucopolysaccharidosis, and these cases, if misdiagnosed, can lead to unnecessary neurological surgery (Ayyildiz et al., 2022). Therefore, it is essential to be familiar with the MRI signal characteristics and location of GTPVS in order to make an accurate diagnosis and avoid unnecessary surgical interventions. PVS have typical MR imaging features; they are round or oval, with well-defined, smooth margins, occur along the path of the penetrating arteries, are isometric with respect to the CSF, and have no enhancement; there is an associated occupying effect in all cases, which varies in severity, and the occupying effect may occasionally lead to obstructive hydrocephalus; GTPVS with occupying effects may present as single or multiple clustered cysts and may be mistaken for malignant disease (Salzman et al., 2005).

In asymptomatic GTPVS, no treatment is necessary and lateral ventricular drainage should be performed when symptoms of hydrocephalus develop. This includes cerebrospinal fluid shunt for hydrocephalus and/or direct hydrocephalus surgery. Postoperative follow-up often reveals recurrence/volume increase (Fujimoto et al., 2012; Woo et al., 2018), so follow-up imaging is essential. There is no standardized MRI follow-up interval for perivascular spaces (PVS), which needs to be determined based on the patient’s clinical presentation, lesion characteristics, and the purpose of the study. The follow-up interval is longer for common PVS, with a median follow-up time of 3.4 years (Li et al., 2024), and may need to be shorter for GTPVS because of their possible greater clinical significance and potential for dynamic change.

## Conclusion

In summary, PVS are a normal physiological component of the CNS. We report a sporadic case of severely dilated GTPVS with an unknown etiology and a non-specific clinical presentation often unrecognized by clinicians. However, the specific imaging features of GTPVS can be distinctive and memorable, and familiarity with and mastery of its imaging manifestations can help radiologists to accurately diagnose and guide the clinical practice, avoiding unnecessary misdiagnosis and inappropriate surgical procedures. In addition, a deeper understanding of the pathogenesis of dPVS and GTPVS may provide valuable insights into the function of the cerebral lymphatic system and cognitive processes.

## Data Availability

The original contributions presented in this study are included in this article/supplementary material, further inquiries can be directed to the corresponding author.
